# The Sociology of Traditional, Complementary and Alternative Medicine

**DOI:** 10.1111/soc4.12182

**Published:** 2014-06-19

**Authors:** Nicola Gale

**Affiliations:** Health Services Management Centre, School of Social Policy, University of Birmingham

## Abstract

Complementary and alternative medicine (CAM) and traditional medicine (TM) are important social phenomena. This article reviews the sociological literature on the topic. First, it addresses the question of terminology, arguing that the naming process is a glimpse into the complexities of power and history that characterize the field. Second, focusing on the last 15 years of scholarship, it considers how sociological research on users and practitioners of TM/CAM has developed in that time. Third, it addresses two newer strands of work termed here the ‘big picture’ and the ‘big question’. The big picture includes concepts that offer interpretation of what is happening at a societal level to constrain and enable observed patterns of social practice (pluralism, integration, hybridity and activism). The big question, ‘Does it work?’, is one of epistemology and focuses on two developing fields of critical enquiry – first, social critiques of medical science knowledge production and, second, attempts to explain the nature of interventions, i.e. how they work. Finally, the article examines the role of sociology moving forward.

## Introduction

Complementary and alternative medicine (CAM) and traditional medicine (TM) are important social phenomena. National and regional studies in the ‘developed’ world have shown high usage of CAM, especially for those with chronic diseases, such as cancer (Molassiotis et al. 2005). In the ‘developing’ world, traditional medicines can be the ‘mainstay’ of health care delivery, particularly in remote or rural areas (WHO 2008, 2013). What is clear is that these phenomena are socially patterned over time and space. Most commentators talk about the rising popularity of CAM, although it is unclear how the recent global economic downturn has affected this, especially given that few TMs or CAMs are available either on national or private health insurance schemes. Any social phenomenon, especially one that generates so much controversy in wider society, is appealing to sociologists. In 1999, Siahpush published a review of this emerging field of study highlighting research in three areas – users of CAM, practitioners and the orthodoxy – and stressing the paucity of empirical research in the field. Over the last 15 years, there has been an explosion of published literature in the field,^1^ and this young sub-discipline is starting to show signs of maturing, evidenced by the growing range and enhanced sophistication of some of the published work in the area.

In this article, I set out to critically review the sociology of TM and CAM, focusing on the last 15 years of scholarship. First, I address the question of terminology, arguing that the naming process itself is a glimpse into the complexities of power and history that characterize this field. Second, I consider the social actors involved in the field. Research on the experiences, attitudes and practices of users and practitioners of TM/CAM has been the empirical foundation on which the sociology of TM/CAM has been built and continues to be refined, developed and theorized. Then, I turn to two newer strands of work, which I term the ‘big picture’ and the ‘big question’. The *big picture* includes concepts that offer interpretation of what is happening at a societal level to constrain and enable the patterns of social practice we see. I start with the concept of pluralism and then move on to explore other interpretations, namely, integration, hybridity and activism. The *big question,* ‘Does it work?’, is one of epistemology, and I focus on two developing fields of critical enquiry – first, social critiques of biomedical science knowledge production and, second, attempts to explain the nature of interventions, i.e. how they work. Finally, I examine the role of sociology moving forward.

## Terminology

Rather than rehearsing all the cases for and against different terminological stances for these medical and healing practices, which may simply result in stalemate, I want to make two overarching arguments about terminology in this field that are relevant for sociological enquiry. First, that naming is an exercise in power that in this field tends to be reflective of ‘Western’ biomedical dominance internationally and, second, that the dualism evident in most terms and definitions used is a product of historical social construction.

Probably the most commonly used term for this group of healing practices in sociology is ‘complementary and alternative medicine’ (CAM), which is a rather indecisive term given that the difference between a state of complementarity, where two things mutually enhance each other's qualities, and positioning a practice as an ‘alternative’ are very different, both practically and sociologically. The absent presence here is, of course, biomedicine; a practice must be alternative or complementary to something. Biomedical dominance continues to frame the language with which we engage with issues of health, illness and healing. Highlighting this is not new; in 1988, Meg Stacey published ‘The Sociology of Health and Healing^1988^’ taking a provocative step by her choice of name for the book to challenge the, relatively uncritical, focus on biomedical practice in medical sociology. In the UK, Cant and Sharma argue that the shift made by the British Medical Association (BMA) from using the term ‘alternative’ to ‘complementary’ in 1993 was motivated by widespread criticism that the BMA was being dogmatic by rejecting non-orthodox approaches completely, so they sought to redefine their role as an ‘arbiter’ of ‘good’ and ‘bad’ medicine. This constituted ‘a shift from the celebration of the cognitive authority of medicine as a form of scientific knowledge to a celebration of its moral authority to protect the consumer by pronouncing standards of competence’ (Cant and Sharma 1999: 104). Holliday (2008) shows how the distinctions between folk/traditional and complementary/alternative have been deployed to reproduce the ‘hegemonic impact of biomedicine on Latino epistemologies’ in the United States. Names are an insight into the social power operating in this field, and acceptance of the terms ‘alternative’, ‘complementary’ or anything ‘other’ to biomedicine could be interpreted as a form of symbolic violence, where individuals misrecognise the domination they experience as natural and inevitable (Bourdieu 1977).

The second point is that dichotomous terms are present throughout this literature. Those studying historical events tend to use the terms ‘quackery’, ‘irregulars’, ‘sectarianism’ or ‘folk medicine’, which are contrasted with the ‘regular’ or ‘orthodox profession’, or ‘allopathy’. Sociological accounts of contemporary society tend to use the terms ‘complementary’, ‘alternative’, ‘heterodox’ or ‘holistic’, to contrast with ‘conventional’, ‘orthodox’ or ‘biomedicine’. The terms ‘folk’, ‘indigenous’ or ‘traditional’ medicine are commonly used to describe long-established, empirically-adapted healing practices originating in a geographically specific area and contrasted with ‘modern’ or ‘professional’ medicine. Another common dichotomy is between ‘Western’ medicine (singular) and ‘other’ medicines – a dichotomy that, apart from reflecting the colonialism implicit in many constructions of medicine, is also historically inaccurate. For instance, practices like osteopathy and homeopathy, while frequently othered, in fact both originated in Western societies (United States and Germany respectively).

While there are clearly differences and variation between medical and healing practices globally, the neat binaries implied in many discussions are problematic. First, definitions change over time, as do alliances. Brindle and Goodrick (2001), for instance, document how over time homeopathy and chiropractic developed very different identities and alliances in the United States and Europe. Second, whichever label is used, it attempts to capture a plethora of practices, products and systems that may bear very little resemblance to each other. There have been numerous attempts to sub-categorize CAM (Ayers and Kronenfeld 2010). Third, just like biomedicine, there is much diversity within, as well as between, practices. Taylor (2004), for instance, explores what she describes as the ‘cultivated misunderstandings’ produced by Chinese medics in response to the influence of Western medicine as they tried to frame their practice for Western consumption. Fourth, in some cases, the actual daily practices being used to heal patients may be very similar across the binary (Johannessen and Lazar 2006). Loeb (2005) describes the management of influenza in Britain at the end of the 19th century and argues that the treatments being offered by regular doctors and those offered commercially (‘patent medicines’ condemned as quackery by the doctors) were very similar. Bivins (2007) and Dew (2000) examine the complex reconfigurations of the practice of acupuncture, which has Chinese roots, by Western-trained doctors in Britain and New Zealand respectively, highlighting the importance of historical and contextual sensitivity in analyses of practice. Finally, the growth of the concept of ‘integrative’ or ‘integrated’ medicine demonstrates concerted efforts by medical practitioners of all persuasions to work collaboratively with others. The challenges and critiques of integration are discussed further below. The binary may still be useful sociologically because as Saks put it in 1995, alternative medicine is a ‘social category’, distinctive by its ‘marginal position in the power relations surrounding health care’ (Saks 1995); however, sociologists should be cautious not to collude with biomedical dominance in the reproduction of these binaries but to remain open and critical by being aware of the productive power of naming and the potential for symbolic violence.

## Users and practitioners of CAM

Much of the early sociological research in the field focused on users. In an early review of the field in 1999, Siahpush argued that many studies had focused on why people use CAM, with six main types of explanation emerging: dissatisfaction with the health outcomes of orthodox medicine; dissatisfaction with the medical encounter/doctor–patient relationship; preference for the way alternative therapists treated their patients, including being caring, individualized attention, ample time and information; the emergence of a new philosophy around nature and holism related to a postmodern value system; the heterogeneity of an individual's social network, resulting in exposure to a wider range of information and values; and finally, that alternative therapies fulfilled a psychological need in the wake of the waning of organized religion, providing an alternative framework for making sense of illness, suffering and misfortune. He suggested that more sophisticated quantitative research, including multivariate analysis, to substantiate these claims was required (Siahpush 1999).

In the last 15 years, there have been substantial empirical developments, including both quantitative studies exploring patterns of CAM usage and qualitative studies exploring patient experiences of using CAM. These have added nuance to our understanding through considering CAM use by different groups in society and in specific contexts. Studies have looked at patterns of usage and motivations for use across populations, for instance, by gender, ethnicity, over time, as well as in relation to health beliefs and wider values, and resourcefulness of individuals' social networks (Arcury et al. 2009; Ben-Arye et al. 2009, 2011; Bhargava et al. 2012; Grzywacz et al. 2007; Heathcote et al. 2011; Kakai et al. 2003; Keith et al. 2005; Leiser 2003; Leung et al. 2005; Lew-Ting 2005; Martinez 2009; O'Callaghan and Jordan 2003; Sarris et al. 2010; Shih et al. 2008; Shiovitz-Ezra and Litwin 2012; Sibbritt et al. 2005; Sirois and Gick 2002; Su and Li 2011; Su et al. 2008; Tanaka et al. 2008; Wiles and Rosenberg 2001). Some studies have employed regression analyses to try and untangle the reasons for observed patterns. For instance, Shippee et al. (2012) found that Black Americans who have experienced racial discrimination (in medical and non-medical contexts) were more likely to use CAM, highlighting perceived barriers to care in institutionalized medical settings. Öhlén et al. (2006) demonstrated that decision-making in CAM was influenced by membership of certain social networks and that ‘becoming a team’ with significant others was important in relation to managing treatment decisions about cancer.

There have also been studies considering experiences of CAM use in specific medical conditions, such as children with Down's Syndrome (Prussing et al. 2005), HIV/AIDS (Foote-Ardah 2003; Pawluch et al. 2000; Thorpe 2008; Torri 2012) and cancer (Bishop and Yardley 2004; Broom 2009; Broom and Tovey 2007a,b; Daykin et al. 2007; Smithson et al. 2010). Other researchers have explored the use of CAM in specific cultural contexts, such as sport (Carter 2010; Kimmerle et al. 2012; Pike 2005), or with particular sub-cultures, such as elder Khmer refugees in the United States (Lewis 2007) or Chinese migrant women in England (Green et al. 2006).

Research on the practitioners of TM and CAM has been less focused on their demographic characteristics, although the gendered division of labor, with many more women involved in practising CAM, has been noted (Taylor 2010). The main focus of the literature on practitioners of CAM has been on the process of professionalization and closely parallels other sociological study of professions (Siahpush 1999). Over the last 15 years, issues of power and the professions have continued to be investigated, with detailed empirical work being carried out to compare and contrast the experience of many different CAM modalities, such as homeopathy, naturopathy or acupuncture, in their professionalization process across the world, including Australia (Baer 2006; Wiese and Oster 2010), Bolivia (Bruun and Elverdam 2006), Canada (Boon 2002; Gilmour et al. 2002; Kelner et al. 2006; Verhoef and Mutasingwa 2006; Welsh et al. 2004), Germany (Sharma 2011), Portugal (Almeida 2012), and the UK (Cant and Sharma 1999; Gibson 2003; Wahlberg 2007). Most of this research takes a neo-Weberian approach (Saks 2003), emphasizing the process of social closure, whereby professional privilege is defended or sought by different groups by restricting other groups' access to resources and rewards. There are some exceptions, such as Clarke et al.'s (2004) Foucauldian exploration of professionalization of nine CAM modalities in the UK. Some professionalization research has focused on the legitimation of the knowledge base of CAM (Hirschkorn 2006), including the reframing of knowledge as scientific (Fadlon 2004a; Tarr 2011) or the deradicalization of knowledge claims (Dew 2000). The knowledge base of CAM is discussed further below.

Providing a theoretical contrast to this literature is historical social research on the professionalization of chiropractic in the United States. Overreliance on the medical model of professionalization, what Brindle and Goodrick termed a ‘survival of the fittest theory’ (2001:569), is highlighted as problematic. Chiropractics in the United States did not seek to emulate medicine; they took the stance that ‘we are not practising medicine at all and we should not want to’ (Brindle and Goodrick 2001:580). They focused on ‘entrepreneurial strategies’ to build up their practice (Villanueva-Russell 2008). Their main client base was in the lower classes, and they made no attempts to convince others about their ‘scientific’ credentials. They remained defiant even when imprisoned for practising medicine without a license. These cases were well publicized in the media, and Brindle and Goodrick argued, this strategy eventually succeeded because ‘the combination of imprisonment, solidarity and media representation turned public opinion in their favor and strengthened their resolve to resist medical domination’ (2001:580).

Siahpush (1999) noted that a future strand of research might be occupational analysis using field research, including issues of socialization, career and occupational communities. Although to date, this has not become a major theme in the research, there are some notable exceptions, looking at education of practitioners (Gale 2008, 2011; Wainwright et al. 2010, 2011) and practitioner communities. Ho (2006) and Hsu (2000) have addressed issues of authority and language in Chinese medicine communities, through examining the ways that the concepts of *qi* (energy) and *shen* (spirituality), respectively, are discursive markers of expertise. Others have discussed the management of tensions in the therapeutic encounter, such as the management of sexuality in bodywork (Oerton 2004), empathy (Ruusuvuori 2005) or discussion of evidence of the effectiveness of homeopathy (Chatwin 2008). There have been some early steps to explore practitioner social networks, such as therapist networks in Mexico (Nigenda et al. 2001), the role of ‘informal networks’ in the UK (Gibson 2003), ‘submerged networks’ in the United States (Schneirov and Geczik 2002), and looking at practitioner identity in relation to wider cross-cultural medical social networks (Zhan 2001). Issues such as career expectations, career trajectories, transitions from education to (usually independent) practice, or the extent to which communities of practice (Lave and Wenger 1991) have developed have not been explored in depth to date.

Increasingly, sociologists have also given attention to the use of CAM/TM by ‘orthodox’ practitioners, including medics, nurses, midwives and pharmacists that are using CAM techniques or practices in their daily work. There have been quantitative investigations, such as a survey of Australian general practitioners that showed how attitudes to CAM become more favorable with younger age, personal experience and patient endorsement (Easthope et al. 2000). However, Hirschkorn and Bourgeault (2005) have pointed out that there has been a tendency to conflate attitudes and behaviors in the literature and so proposed a new conceptual framework, where the ‘outcome’ was provider decision over whether or not to use CAM or refer to CAM practitioners. They argued that this outcome is influenced by the personal and professional characteristics of the provider, the personal characteristics and disease state of the patient, the physical and organizational structure in which they were working, and the epistemological and political status of the CAM modality under consideration. Other research has considered how professional identity is shifted and asserted in relation to the adoption of CAM practices, as a way to emphasize ‘care’, ‘holism’ or ‘health’ (Baer 2008; Dew 2000; Gilbert 2004; Tovey and Adams 2003; Winnick 2006), but it has been recognized that the impact of this on practice has been constrained by the continued authority of biomedicine and the medical profession (Cant et al. 2011).

In studies of practitioners, the dichotomy between ‘orthodox’ and ‘other’ is often destabilized, even when not explicitly critiqued. Some researchers have actually focused on the process of construction, maintenance and communication of the dichotomy. The concept of ‘boundary work’, from actor network theory, has commonly been elaborated to explore this (Derkatch 2008; Keshet 2009, 2011; Norris 2001; Shuval and Mizrachi 2004; Shuval et al. 2002), demonstrating that the everyday practice of human actors within the field produces the boundaries:

Weak assertions [“we are scientific”, “we are holistic”] become hard facts as they find their way into textbooks, lectures and publications. In this way, they become symbolic of particular groups and organisations, and ultimately such representations come to be viewed as reflective of the natural order (Broom 2002:233).

The numerous studies of the characteristics, experiences and practices of social actors in the field of medicine offer insights into the construction of the concepts of CAM and TM (used generally in line with a broad North/South and West/East geographical divide) and the deployment of power at the micro-level in clinical practice, and provide the building blocks for consideration of wider societal trends.

## Big picture

As the sociology of CAM and TM has matured, researchers have increasingly tried to make links to wider theories of societal change, drawing where appropriate on historical research, and to be more theoretically rigorous. I will first examine the concept of medical pluralism, which has been for many years the most widely adopted ‘big picture’ concept and then look at three emerging concepts: integration, hybridity and activism. For each concept, I will examine the structural forms that they describe, the social trends they relate to, and relate these back to how they are negotiated at a micro level by social actors.

Medical pluralism draws attention to the diversity of practices in a health system and can be distinguished from medical monism where there would be unity of practice or medical dualism where there would be a binary opposition of practices. Published in 1999, Cant and Sharma's seminal monograph, ‘A new medical pluralism’, reignited the debate about pluralism. They pointed out that medical pluralism has a long history. In the UK, it had its roots in the purveyance of patent medicines in the 19th century, which the regular profession fought hard to wipe out because it was a challenge to their hegemony (Loeb 2005; Porter 1989). More recently, there had been a revival of pluralism in the form of increased use, practice and popular legitimacy of CAM, with similar resistance evident from the medical profession. Criticism is often couched in terms of concerns for patient safety (either directly because of the risks of harm through CAM treatment or indirectly through risks associated with avoidance or delay in seeking ‘orthodox’ medical assistance) (Ernst et al. 2004). Cant and Sharma argued, however, that in this ‘new’ pluralism, all practices were not present on an equal basis but that biomedicine retained dominance. Nevertheless, there was an important disruption of the division of medical labor. The State, they argued, had a responsibility to address issues of funding and regulation. Since then, research explicating medical pluralism has drawn on this work, considering issues such as the disclosure of CAM use in medical consultations (Stevenson 2004), the use of acupuncture by medical doctors (Dew 2000), the recasting of regulatory and ethical debates on quackery (Wahlberg 2007) and the importance of acknowledging pluralism when designing ‘culturally sensitive’ healthcare systems (Green et al. 2006; Kiesser et al. 2006). Some have argued that the focus on clinical legitimacy and market demand in medical pluralism is illustrative of postmodern and relativist values, including a ‘return’ to and commodification of nature and spiritual values (Eastwood 2000) and the decline in civic culture and substitution of individual self-improvement (McQuaide 2005).

An important strand in this work has been the ways in which pluralism is embodied and enacted by the users or consumers of medical care who, some have argued, tend to make choices based on pragmatism and perceived therapeutic effectiveness rather than philosophy or scientific evidence (Broom and Tovey 2008; Chacko 2003; Connor 2004; Fadlon 2004b; Little et al. 2007; MacArtney and Wahlberg 2014). Users have been described as ‘bricoleurs’, drawing on Levi-Strauss (Broom 2009; McClean 2005). This literature points less toward a postmodern society but a late modern one. As Broom puts it:

Rather than engendering the loss of metastructures and pursuit of pure individuation, bricolage is intended to engender individual agency/action within competing structures of health knowledge and the activity of pragmatic (but structurally constrained) individuation (Broom 2009: 1032).

However, some have argued that we have moved ‘beyond’ both dualism and pluralism to a ‘grudging acceptance’ then ‘integration’ of CAM and ‘orthodox’ medicine (Broom and Tovey 2007b; Wiese et al. 2010). It is difficult to overestimate the proliferation of debate in this area, and much of it has been in the clinical and applied health research literature; however, sociological analyses are also common. In a review by Wiese et al. (2010), the authors argued that the mainstream inclusion of CAM has happened in three distinctive ways: pluralization, incorporation and integration. The first, pluralization, means that each practice retains its distinctiveness and, as discussed above, the user often performs a consumer role selecting the approach most suited to them. The second involves the selective incorporation of aspects of CAM to be used alongside biomedical treatment. This can be in the form either of CAM techniques learned by ‘orthodox’ HCPs (as discussed in the previous section) or by CAM practitioners working alongside ‘orthodox’ HCPs but in a contained way where the latter operate at gate keepers. The final one, integration, involves respect and collaboration between different views of health and healing, resulting in mutual transformation. The important distinction between integration as incorporation (with asymmetry of power favoring biomedicine and the orthodox professions) or as mutual transformation (with more symmetry and genuine complementarity) is not always acknowledged in the debates about integration. ‘Integration’, therefore, runs the risk of becoming a conceptual dead end without more selective and critical deployment. There is another potential issue with the term: it is now being regularly used in the wider health services literature to describe the process of integrating health and social care, rather than different types of healthcare (Glasby 2005).

There have been a number of micro-sociologies of ‘integrative’ healthcare settings that almost all point to a dualist conception of medicine and an incorporation strategy that maintains the dominance of biomedicine. Shuval et al.'s study showed that in the four integrative clinics in Israel they studied, biomedical practitioners focused on the diagnosis and treatment of disease, while ‘alternative practitioners work in the illness context, concentrating on feelings and affective states involving the alleviation of pain, suffering and efforts to improve the quality of life’ (2002:1745). This has echoes of the gendered division of labor between medicine and nursing (Witz 1990). Hollenberg studied two newly established integrative clinics in Canada and found that

biomedical practitioners enact patterns of exclusionary and demarcationary closure, in addition to the use of “esoteric knowledge”, by: (a) dominating patient charting, referrals and diagnostic tests; (b) regulating CAM practitioners to a specific “sphere of competence”; (c) appropriating certain CAM techniques from less powerful CAM professions; and (d) using biomedical language as the primary mode of communication. CAM practitioners, in turn, perform usurpationary closure strategies, by: (a) employing their own “esoteric knowledge” in relation to biomedicine and other CAM professions; (b) appropriating biomedical language and terminology; (c) increasing their professional status by working with biomedicine; and (d) referring among CAM practitioners to increase patient flow (Hollenberg 2006:731).

The positionality, power and constraints of actors in the integrative setting are also important. Keshet (2013) interrogated the structure–agency dynamic in relation to the embeddedness of actors in orthodox medical institutions and developed the concept of ‘dual embedded agency’ to explain how practitioners that practised both biomedicine and CAM were uniquely able to achieve change using ‘non-adversarial’ strategies.

Links between different integration strategies and wider patterns of power have been noted. Hollenberg and Muzzin (2010) argued that anti-colonial theory can aid our analysis of the appropriation of indigenous knowledge and the enduring dominance of biomedicine. The logical conclusion to the incorporation approach to ‘integration’ is the full incorporation of competing practices. Sharma (2011) has documented how this happened with lay practitioners of Naturheilkunde in Germany and their process to becoming ‘Physical and Dietary Therapies’ during the interwar period. Many of the debates on integration seem more rooted in modernity, rather than late or postmodernity. This has produced some critical scholarship, which indicates that the collaboration of CAM and orthodox professionals is not surprising in a market economy and argue that this convergence to a medical monism is not a real alternative to modernity. Hsu (2000) showed how Chinese medicine in Tanzania flourished through entrepreneurial activity. Nisula (2006) described the integration of Ayurveda in the context of a market economy in Mysore, India. Tillman (2002) looked at the role of health insurance companies in the integration process. Ning (2013) describes how concepts such as holism are deployed very similarly across the orthodox/alternative binary and that both are shaped to meet consumer needs. Han argued that

In contemporary capitalist societies the key structuring/generative mechanism affecting the form and content of medical systems derives from the commodification of health care. This pervades both orthodox medicine and its various alternatives. (Han 2002:1).

Following these lines of argument, integrative strategies are more likely to maintain modernist and colonial structures and perpetuate social inequalities rather than challenge them. Scott (1999) made the distinction between wider-self holism and wider-world holism, arguing that CAM tends to focus on the former, bringing notions of mind, spirit and self into healing practices but not that of societal structures and environment that characterize a (critical, rather than medical) public health approach (Givati 2012). All this results in a ‘passive’ rather than ‘active’ form of consumerism in integrative medicine (Frank and Stollberg 2004b).

Patient experience research offers a challenge to some of these arguments by suggesting that integrative medicine can be experienced as empowering and ‘indicative of a shift from a hierarchical to a more collaborative relationship’ (Hök et al. 2007:1642). In their review of cancer patients experiences of using CAM, Smithson et al. found that ‘experience of integrated systems, or of a unified health service offering biomedicine and complementary therapies options, was almost universally appreciated, and these services facilitated a positive experience for participants’ (2010:32). This has parallels with the wider literature about partnership and shared decision-making (Gale 2008). However, Tovey and Broom's (2008) research cautioned that use of CAM should not be conflated with advocacy for integrated healthcare. Other issues such as risk, evidence, cost, location of provision and the epistemological identity of providers are relevant to users and mediate their views on the appropriateness of integration.

The concept of ‘hybridity’ is rooted in wider notions of a globalized society and within post-colonial scholarship, where hybridization is contrasted with homogenization or polarization (Holton 2000). The term has been generally used in a positive way, to refer to a ‘playful combination of various cultural elements in art and literature, and particularly to the forming of an identity in the context of migration’ but has only recently been applied to medicine (Frank and Stollberg 2004a). There have been a number of studies applying this theoretical perspective to empirical data on movements of practices across national and cultural boundaries (Kim 2006; Obadia 2007; Reddy 2002; Santosh 2013). Using hybridity to interpret the ‘big picture’ offers two distinctive contributions to the sociology of TM and CAM: first, it offers a more spatially-informed analysis, and second, it is an alternative to the ‘integration’ concept (that is less Western-centric).

Hybridity offers a spatial or territorial dimension to analysis giving opportunities to look at how power and knowledge are enacted and re/produced in different geographical and cultural locations. For instance, researchers have considered the process of cross-cultural translation of medical concepts (Garvey 2011; Kim 2006; Reddy 2002; Sagli 2010) as well as documenting the ways that transfer, exchange and transformation of knowledge happen (Scheid 1999, 2002). Khan argues that a simple biomedical dominance model is insufficient to explain the medical system in India and that, ‘although the “cultural authority” and hegemony of biomedicine over indigenous science and knowledge were initiated by the colonial state, they were extended by the mainstream national leaderships and national governments with far more extensive and profound implications and less resistance’ (Khan 2006:2786). Reddy (2002) explores how Ayurveda in the United States has interacted not only with the American medical culture but also with ‘New Age’ spiritualities, and Obadia (2007) has described how the religious practice of Buddhism has become ‘therapized’ in France.

The concept of hybridity challenges the empirical basis of both pluralism, which implies little merging of philosophy and practice and can overlook political dimensions (Broom et al. 2009), and dualism, which constructs an illusion of incommensurability (Keshet 2011; Stevenson 2004). Hybridity offers an alternative to accounts of integration that tend to stress the ‘dominance’ of biomedicine and are, therefore, too blunt to explain empirical data that capture the diversity and creativity of hybrid forms and practices. Hybridity does not sidestep issues of power; indeed, some of the debate on hybridity echoes (using different terms) previous discussion about power and the comparisons between pluralism, monism and dualism (Frank and Stollberg 2004a), and post-colonial scholarship is rooted in analysis of global power relations. There are related theoretical interpretations, such as Wiese et al.'s (2010) concept of transformatory integration or Fadlon's (2004a) concept of domestication, a process by which ‘the foreign is rendered familiar and palatable to local taste’, but both of these are explicitly focused on Western cultures. Post-colonial analysis upturns traditional theories of medical and colonial power by making space for resistance (Wahlberg 2006). Johnston argues that the use of CAM can be much more than a pragmatic, apolitical pluralism:

Indigenous medicine provides a vehicle through which to express individual and cultural identities and to take a stance in relation to a history of colonization and ongoing power relationships with the dominant society. Outmoded concepts like a simple dichotomy between traditional and modern get resoundingly upended by the realities in native communities (Johnston 2002:209).

This links to the final ‘big picture’ model, which I have termed activism. One of the primary functions of medical power is the control of bodies (Turner 1995), so resistance to modernist power structures is logically also an embodied form of resistance. Empirically, there are links between use of CAM and participation in social movements, such as the women's, social justice, community development and green movements (Gibson 2003; Scott 1998), lifestyle reform movements, such as vegetarianism and anti-vaccination (Adams forthcoming) and anti-pharmaceutical activism, although clearly not all CAM users would share these political views.

Some sociologists have critically explored the extent to which CAM practices can be linked to these forms of political resistance (Gibson 2003; Hess 2002). For instance, Scott (1998) explores whether homeopathy can be considered a feminist form of medicine. Napolitano and Flores in their research on the use of Chinese medicine in Mexico have argued:

Some forms of oriental medical diagnosis and cure are creatively translated into a grassroots form of medicine which, in its everyday form of practice, can partly challenge gender position and reformulate a power of a “national” culture, even if, in practice, some of the efficacy of oriental medical forms may be diluted (2003:90).

I would argue that a key contribution of the activism ‘big picture’ approach is to challenge the assumptions sometimes implicit in the ‘integration’ concept, i.e. that being more like biomedicine is the goal and that formal networks are key (Brindle and Goodrick 2001; Gibson 2003; Schneirov and Geczik 2002). However, like any form of activism, there is often a tension between staying true to core values (and awaiting revolutionary change) and the potential for faster change through collaboration and compromise with those in power (Goldner 2001; Villanueva-Russell 2011). There is potential to develop this big picture concept much further by drawing on interactionist sociology and pursuing networked explorations of social movements and healthcare practice.

## The big question

Probably the most ubiquitous question in wider society about CAM is ‘Does it work?’ It is tempting for sociologists to say that they are not interested in answering that question; that it is enough that it is a major social phenomenon and that users experience it as effective, perceive it as valuable and so continue to use it, to make it worthy of study. However, more recently, sociologists have shown more willingness to engage critically with these publically ‘untouchable’ questions of medical knowledge and science (Barry 2006) and, by drawing on fields such as science and technology studies, cultural studies, anthropology, religious studies and embodied sociology, have started to explore and construct alternative knowledges about *how* CAM might work, i.e. what the nature of the therapeutic intervention is. Nonetheless, these voices remain nascent within the overall picture of the sociology of CAM.

The most well developed part of this set of arguments is that the claims of scientific truth and unity by decriers of CAM (i.e. the claim that medicine is based on evidence and practices are only alternative because they cannot be or have not yet been scientifically proven, and if they are proven, they will cease to be alternative and will be part of medicine) are untenable sociologically (Keshet 2009), not least because the model of evidence they use is based on Western biomedical notions of truth. This myth of scientific unity could be considered part of a calculated exclusionary strategy; studies on the professionalization of CAM have frequently considered the responses of the medical profession (Kelner et al. 2006; Winnick 2005). For instance, Winnick studied the American medical profession and argued that although there have been phased changes over time (from condemnation (60s–70s) to reassessment (mid 70s–early 90s) and to integration (90s)), biomedicine has retained its dominance, and that ‘dominance is sustained through adaptation to structural change’ (Winnick 2005: 38). In the integration phase, she argued that the primary means of control was the subjecting of CAM to scientific scrutiny.

The myth may, however, also be a result of asocial naivety: concepts of science are developed through lay and professional socialization processes and may not be reflexively considered by social actors. These unchallenged assumptions about the world and knowledge are what Bourdieu (1977, 1990) would term ‘doxa’, and they produce the potential for symbolic violence, discussed in the Terminology section above. Sociologically, the very existence of boundary work in the field of scientific knowledge production (Keshet 2009) and the ability of the structures of orthodox medicine to co-opt potentially dissenting voices from the lay public and CAM practitioners (Hess 2004) are clear signs that science and non-science labels are constructed socially. The mixed views of CAM practitioners on the growth in randomized-controlled trials to investigate the efficacy of various CAM treatments are also illustrative of the social construction of scientific knowledge. Some have resisted biomedical forms of evaluation whereas others believe that these will bring greater legitimacy to their practices (Jackson and Scambler 2007). Given that historical sociology has clearly demonstrated the adaptive resilience of biomedicine (Timmermann 2001), the repeated co-option of parts of CAM and the production through biomedical power of an ‘autonomous individual of transnational, neoliberal governance’ (Fries 2008:353), it may be naive to think that these evaluation methods alone could be enough to confer legitimacy.

This leads onto the second (theoretical) dimension in the ‘big question’ arguments: the importance of embodiment and intersubjectivity. A classic argument in the decrier camp is that CAM is ‘just placebo’. The proponents of CAM counter with claims about alternative conceptions of the body, be it *qi*, *shen*, vital force or energy, arguing that these things cannot be reduced to biomedical notions of reality and must be altered or balanced to achieve healing. The sociological scrutiny of both ‘sides’ of this argument has been limited, but there are some promising developments that deserve further research. To quote Wahlberg, an anthropologist:

If pharmacologists and clinicians have corporeally located the concept of efficacy in terms of bio-availability, pharmacodynamics and pharmacokinetics, and herbalists in terms of a herbal revitalizing of the body's own vis medicatrix naturae, from the early 20th century onwards medical anthropologists (especially those who became interested in the ‘savage mind’) have built up an equally rigorous theory of symbolic efficacy in terms of narratives, symbols and a kind of cognitive homeostasis (Wahlberg 2008:177).

Hence, there is no ‘just’ about placebo; it is a complex and embodied social process. Sointu (2006a) uses the concept of recognition and argues that gaining recognition through intersubjective interaction for one's unique individuality can be part of a healing process, within ideas of well-being that go beyond physical function. Gale (2011) developed the concept of body-stories, bringing together notions of the body, narrative and space, to describe how the palpatory or energetic touch of the investigation part of osteopathic and homeopathic consultations respectively, in combination with listening and observing, can be the first stage in the healing process. Barcan, through a Foucauldian lens, explores narratives of the self as therapeutic tools and suggests that ‘if we argue that power operates in and through the body, then perhaps it takes bodily practices to help us *undo* that power’ (2008:25, emphasis in original; see also Schneirov and Geczik 1998).

Another dimension of placebo is that belief in the effectiveness of a treatment can itself cause the effect, yet the pragmatic approach of many users does not necessarily support that argument. From the methodological perspective, it is difficult with cross-sectional studies to know whether ideological beliefs, such as ‘postmodern values’, are the cause or the consequence of the use of CAM (Siahpush 1999). With a cohort study, it may be possible to identify patterns of causality to this relationship more convincingly. However, ethnographic approaches have brought insights. While the studies on social movements reviewed above suggest that political beliefs may lead to CAM, Baarts and Pedersen (2009) have explored why people continue to use CAM after the symptoms they initially presented with have gone. They argued that users get ‘derivative benefits’, including ‘a fresh and sustained sense of bodily responsibility that induces new health practices’ (Baarts and Pedersen 2009:719). The concept of responsibility and its role in the healing process has caused some divergent views in the literature, often drawing on the work of Rose (1990). On the one hand, some sociologists have argued that CAM contributes to victim-blaming, or ‘holistic sickening’ (Sered and Agigian 2008), with others arguing that it also has the potential to promote creativity, innovation and empowerment (McClean 2005), that self-responsibility and self-actualization are intrinsic to the sense of subjective well-being many CAM users are seeking (Sointu 2006b) or that it can be part of attempts to reestablish a sense of personal control after illness (Lee-Treweek 2001).

However, many practitioners argue that there is more to their treatment than the intersubjective effects of the therapeutic encounter. There have been sociological discussions of how practitioners and lay people construct expertise in CAM (Hirschkorn 2006; Pedersen and Baarts 2010) in addition to the professionalization literature. However, some researchers have attempted to interrogate the discourses of TM and CAM. While some operating within, what I have termed, the ‘big picture’ model of integration have questioned whether these discourses are genuinely alternative (Ning 2013), others have explored them on their own terms, often ethnographically, including notions of energy medicine (Keshet 2011), vitalism (Villanueva-Russell 2005) or meridians and chakras (Fadlon 2004a). Three contributions are worthy of particular note: the first, methodologically, and the other two, theoretically. Tarr's (2008) auto-ethnographic approach to exploring the Alexander technique brought an original and insightful perspective to understanding the embodied and social nature of the therapeutic intervention. In Johnston and Barcan's (2006) investigation of the ‘subtle body’, which ‘figures the self as multiple, extensive and radically intersubjective’, they argued that consideration by cultural studies of these *ex vivo* concepts could potentially enrich and expand the discipline's theoretical basis. Finally, Scott's (2003) sociological reading of homeopathy argued that it may work by sitting on a ‘borderland’ of the social and biological worlds.

## The future of the sociology of CAM and TM

Over the last 15 years, sociologists have continued to engage critically with CAM and TM and contributed numerous empirical studies of the social phenomenon. The focus has been much more strongly on qualitative rather than quantitative methodology, in contrast to many of Siahpush's suggestions for further research in 1999. Looking forward, I would argue that there are a few directions that sociologists could go. Burawoy (2005) makes the distinction between policy, professional, critical and public sociology, and each of these sociologies potentially has a part to play. Policy sociology relates to the deployment of sociology in the pursuit of policy goals and may include national efforts to regulate TM/CAM professions or moderate access. It could also be interpreted as sociology *in* TM/CAM through efforts to support practitioners, professional bodies and educators in their goals to improve the quality of care. It may include work on interprofessional education (Shneerson et al. 2013; Willison 2008) or collaborations with practitioner-researchers and clinical scientists. Increasingly, universities are indeed being assessed on their ability to achieve ‘impact’ with their research. Professional sociology is the dispassionate investigation of social phenomena and continues to have an important place by describing and understanding CAM use and practice. Here, there may be particular scope for interdisciplinary working where appropriate (such as for understanding how CAMs work) and more creative methodological approaches (particularly around embodiment). Critical sociology aims to explore *why* social phenomena are as they are and to uncover patterns of power and injustice. Here particularly, the challenge to avoid reproducing biomedical dominance in terminology and analysis is vital. Critical perspectives have long been a strong strand of medical sociology and similar analysis can be applied to TM and CAM as conventional medicine. A key question might be: to what extent does CAM contribute to or challenge health inequalities? Finally, public sociology would seek to use sociological insights to stimulate public discussion about our health and illness,^2^ our bodies and our health system. There are not likely to be any major concessions from biomedicine or a dismantling of the medical profession, given its adaptive resilience, but there may be scope for sociologists working in a critical friend role with activists globally who are demanding health systems to be more responsive to their health needs.

## Footnotes

1 I found over 250 books and peer-reviewed articles in sociological journals (or taking a sociological approach) published after 1999. *Social Science and Medicine, Health: Social History of Medicine* and *Sociology of Health and Illness* were the most prolific publishers of articles on the sociology of CAM. I searched systematically for the articles, using search terms ‘complementary and alternative medicine’ or the other terms covered in the ‘terminology’ section of this article or names of specific CAM modalities (such as homeopathy and acupuncture) and sociology. I only reviewed articles published in English, which limited the international scope of the review; in particular, while I did include some, I very likely missed valuable studies around use of CAM and TM in China, Japan, Vietnam, South Korea and India.

2 I have given a number of public and educational talks on sociology and CAM, including a debate on whether CAM should be regulated (http://www.birmingham.ac.uk/research/impact/perspective/debate/medicine.aspx) and a presentation at *Cafe Scientifique*, which holds talks on various aspects of science in the pub.
